# Social stratification factors in educational transitions: a comparative analysis of Argentina, Chile and Mexico

**DOI:** 10.3389/fsoc.2026.1764458

**Published:** 2026-04-29

**Authors:** Paula Boniolo, Sebastian Lemos

**Affiliations:** 1 Instituto de Investigaciones Gino Germani, Facultad de Ciencias Sociales, Universidad de Buenos Aires, Buenos Aires, Argentina; 2 CONICET, Buenos Aires, Argentina

**Keywords:** educational research, educational expansion, educational inequalities, educational stratification, educational transitions

## Abstract

**Background:**

Educational expansion in Latin America has not necessarily led to greater equality of opportunity.

**Methods:**

This study analyzes educational transitions in Argentina, Chile, and Mexico using nationally representative surveys and logistic regression models with average marginal effects.

**Results:**

Findings show that inequality persists but is reorganized across stages depending on institutional configurations.

**Conclusion:**

Educational systems reproduce inequality through differentiated pathways, highlighting the need to consider institutional design beyond expansion policies.

## 1 Introduction

Latin America remains one of the most unequal regions in the world, and education plays a central role in both the reproduction and transformation of those inequalities. Over recent decades, the expansion of educational systems—particularly at the secondary and tertiary levels—has been associated with expectations of democratized access and increased social mobility. However, available evidence indicates that the relationship between educational expansion and equality of opportunity is neither automatic nor uniform.

This article analyses inequalities of educational opportunity in Argentina, Chile, and Mexico around 2020 through a comparative approach focused on educational transitions, from primary school completion to access to and graduation from higher education. Rather than concentrating exclusively on final attainment, the analysis examines critical turning points along educational trajectories in order to identify the stages at which opportunity loss is most pronounced. Three classic dimensions of stratification are considered: social class of origin, household educational climate, and gender.

The selected countries represent contrasting institutional configurations. Argentina combines tuition-free access, strong state provision, and wide coverage; Chile has consolidated a highly privatized and segmented system; and Mexico exhibits uneven expansion shaped by persistent territorial disparities. These arrangements allow for an assessment of how different education regimes mediate the impact of social origin on schooling trajectories.

Regional research documents sustained growth in secondary schooling and more gradual expansion of higher education (Perosa et al., 2021), without a corresponding reduction in educational and labor-market inequalities (Alcoba, 2014; Zenteno and Solís, 2006). Comparative studies show that expanded access has coexisted with persistent stratification across both transitions and final outcomes (Solís, 2018). In Argentina, despite institutional growth, substantial inequalities remain in secondary completion and higher education graduation (Boniolo et al., 2025a,b; García de Fanelli, 2014; Jorrat, 2010). In Chile, the market-oriented expansion of higher education has deepened institutional segmentation and reinforced social selectivity (Espinoza and González, 2012; Jorrat et al., 2024). In Mexico, socioeconomic background, parental education, and territorial disparities continue to shape educational continuity (Pereyra, 2008; Solís, 2013, 2018).

From a theoretical perspective, the article engages with the main hypotheses on educational stratification: social selectivity over transitions (Mare, 1980), maximum maintained inequality (Hout and Rafftery, 1993), effectively maintained inequality (Lucas, 2001), and inequality linked to system coverage (Solís, 2013, 2018). Together, these frameworks make it possible to assess whether expansion operates as a mechanism of equalization, reproduction, or transformation of social inequality.

Empirically, the analysis draws on nationally representative probability surveys: ESAyPP/PISAC-COVID (2021) for Argentina, ENOP-CEP (2019) for Chile, and the Intergenerational Social Mobility Module of INEGI (2016) for Mexico. Transition rates and logistic regression models with average marginal effects are estimated, allowing for cross-national comparison of the magnitude of stratification factors at each educational transition. The article makes a twofold contribution: it provides recent comparative evidence on three institutional configurations within the region and adopts a transition-based approach that allows for more precise identification of the critical nodes in the reproduction of educational inequality.

This study makes three main contributions. First, it provides comparative evidence on three processes of educational expansion across three Latin American countries. Second, it adopts an educational transition–based approach, which allows for a more precise identification of the moments—or critical junctures—at which inequalities emerge, intensify, or are reconfigured along educational trajectories. Third, it integrates the analysis of educational expansion with the specificities of each educational system, showing that similar levels of expansion may give rise to differentiated patterns of inequality depending on system-level characteristics. Taken together, these contributions generate relevant evidence for the design and evaluation of educational policies aimed at mitigating inequalities in Latin American countries.

## 2 Developments in the field of educational inequality in Argentina, Chile, and Mexico

Educational expansion in Latin America has historically been conceived as a central mechanism for upward social mobility and for improving living conditions. However, empirical evidence accumulated over the past decades shows that this relationship is neither linear nor automatic. Since the 1980s, secondary and tertiary education have expanded throughout the region, albeit with different rhythms and institutional configurations. Research on educational trajectories in Latin America shows that the increase in secondary completion and the gradual growth of access to higher education have been relatively consistent trends, particularly in countries such as Argentina, Chile, and Uruguay (Perosa et al., 2021). Nevertheless, this process has not translated into a systematic reduction in inequalities in educational and labor-market opportunities (Alcoba, 2014; Zenteno and Solís, 2006).

One key explanation for this phenomenon is that, although education has consolidated its role as a mediator between social origins and class destinations, this mediation has not produced a clearly equalizing effect. In other words, while social origin increasingly shapes occupational outcomes through educational attainment, the expansion of access alone has not been sufficient to offset initial disadvantages. Comparative studies consistently point out that access expansion has coexisted with persistent stratification in both educational opportunities and final achievements (Solís, 2018).

In Argentina, educational massification developed within a predominantly public and tuition-free system, historically associated with upward mobility for the children of migrants in the first half of the twentieth century. However, recent studies indicate that, despite institutional and territorial growth, class inequalities and family cultural capital continue to exert a strong influence on both secondary completion and higher education graduation (Boniolo et al., 2025a; García de Fanelli, 2014; Jorrat, 2010). In addition, retention problems and academic performance disproportionately affect working-class youth and those living in less developed regions, reinforcing gaps in effective access to credentials (Boniolo et al., 2025b).

The Chilean case has been widely examined in the literature on intergenerational mobility. Some studies argue that individuals whose parents attained higher education are more likely to reach the same level, and that these inequalities have declined among younger generations (Torche and Wormald, 2004). However, subsequent comparative analyses complicate this interpretation, showing that Chile exhibits higher levels of inequality of educational opportunity, lower returns to schooling, and a limited capacity of the education system to attenuate the link between origins and destinations compared to Argentina and Mexico (Solís and Dalle, 2018). These outcomes have been attributed to the advanced commodification of access to higher education and the deepening segmentation between public and private sectors. In this context, higher education expansion took place within a privatized system organized around demand-side financing and characterized by sharp institutional differentiation, reinforcing social selectivity and quality disparities between institutions (Espinoza and González, 2012; Jorrat et al., 2024).

In Mexico, studies conducted since the second half of the twentieth century documented a strong association between social origin and educational attainment, albeit with a gradual downward trend. Balan et al. (1977) showed that education was the most decisive factor in occupational attainment, even more influential than direct effects of social origin. Later research identified increased intergenerational educational mobility, followed by a slowdown among more recent cohorts (Binder and Woodruff, 2002; Torche, 2010). Although the present study does not directly address mobility, these findings underscore the persistent influence of educational origins on labor trajectories. Educational expansion in Mexico has also been accompanied by enduring processes of social differentiation. Household socioeconomic background, parental education, and access to cultural and territorial resources remain decisive for educational continuity and achievement (Blanco Bosco, 2011; Solís, 2013, 2018). Inequalities are expressed both in the transition from secondary to higher education and in the horizontal stratification of the system, where the choice between public and private institutions functions as a mechanism for the reproduction of class differences (Pereyra, 2008).

Recent research on educational inequality in Latin America and Southern Europe confirms the persistence of origin-based disparities even in contexts of sustained expansion. Solís (2018) shows that in countries such as Argentina, Chile, Mexico, Spain, and Portugal, inequalities concentrate in secondary completion and tend to decline only at the early stages of expansion, thereby challenging both the social selectivity hypothesis and the maximum maintained inequality hypothesis. In Mexico, Rodríguez (2020) identifies structurally upward educational mobility accompanied by persistent barriers that vary by gender and cohort. Studies compiled by López and Rodríguez (2022) further document the segmentation of educational trajectories and the tension between agency and structure, emphasizing the role of social and territorial background in access and persistence. Finally, Solís and Dalle (2018) show that education continues to operate as a mediator between origins and destinations, albeit with limited equalizing capacity. In this vein, research by Boniolo and Lemos (2025) shows declining probabilities of secondary and tertiary graduation in recent Argentine cohorts, with marked regional differences. Similarly, studies by Lemos (2024, 2025) show that the type of secondary school attended is a critical factor shaping educational advancement and labor-market entry.

In addition, the literature on educational inequality in Latin America consistently points out that the expansion of educational systems, while broadening access, has not led to a drastic reduction in inequalities of origin. On the contrary, numerous studies show that inequality tends to be reorganized along educational trajectories, becoming concentrated at different stages depending on the structural characteristics of each system (Alcoba, 2014; Solís, 2018; Zenteno and Solís, 2006). Within this framework, the analysis of educational transitions has been identified as a key strategy for understanding the stages at which inequalities emerge, intensify, or are reconfigured (Lucas, 2001; Solís and Blanco, 2018; Solís, 2013, 2018). Available evidence indicates that in Mexico the main bottlenecks are located at early stages, particularly in the completion of secondary education (de Olguín Meza, 2018; Solís, 2013); that in Chile inequalities are concentrated in access to higher education within a highly segmented system, even though intergenerational improvements are observed at earlier stages (Espinoza and González, 2012; Jorrat et al., 2024; Torche and Wormald, 2004); and that in Argentina, despite relatively broad access, gaps shift toward persistence and graduation at the tertiary level (Boniolo et al., 2025b; García de Fanelli, 2014; Jorrat, 2010).

Taken together, the evidence from Argentina, Chile, and Mexico reveals differentiated institutional trajectories leading to convergent outcomes: educational expansion has increased absolute opportunities for access, but it has not eliminated relative inequalities. Comparative literature (Dalle et al., 2019; Solís, 2018) consistently shows that ascribed factors such as social class, parental education, gender, and region of residence continue to shape educational attainment and effective access to higher education.

## 3 Ascribed factors and inequality of educational opportunity: theoretical framework and hypotheses

The analysis of inequality of educational opportunity is grounded in the relationship between social origins and educational outcomes. Within the sociology of stratification, education is understood as an intermediary mechanism between the class position of the family of origin and individuals' occupational destinations. The strength of this relationship depends on the level of development of the educational system, its institutional organization, and the scope of access and inclusion policies in each country (Blau and Duncan, 1967).

International literature has developed a set of hypotheses to explain under what conditions educational expansion does—or does not—lead to a reduction in origin-based inequalities. These approaches were originally formulated in early industrialized societies and have later been revisited in light of more recent experiences, such as those of Latin America, where higher education expansion took place in contexts of strong institutional segmentation and persistent structural inequality (Solís, 2018).

The hypothesis of social selectivity over transitions (Mare, 1980) holds that the influence of social origin is stronger at early stages of the educational trajectory and tends to decline as individuals advance to higher levels. From this perspective, working-class students who manage to access higher education constitute a socially selected group with unobserved attributes—such as stronger motivation, higher educational expectations, or greater family support—that partially compensate for their structural disadvantages. As a result, inequality is expected to decrease at advanced stages of schooling.

By contrast, the hypothesis of maximum maintained inequality (Hout and Rafftery, 1993) argues that coverage expansion does not necessarily lead to a reduction in class differentials. During early expansion, upper and upper-middle classes capture most of the new educational opportunities, sustaining high levels of inequality until saturation is reached. Only thereafter do middle and working-class groups gain broader access, so inequality remains high during the initial stages of system growth.

The hypothesis of effectively maintained inequality (Lucas, 2001) introduces a distinction between vertical and horizontal inequality. Even when differences in attained levels of education decline, inequality persists through qualitative differentiation among institutions, fields of study, and credentials. Institutional segmentation and diversification thus enable higher-status groups to retain access to the most prestigious educational pathways.

From Latin America, Solís (2013, 2018) proposed the hypothesis of inequality linked to coverage, which emphasizes the structural role of educational systems in the reproduction of inequality. In contexts of limited expansion and weak compensatory policies, absorption rates remain insufficient to incorporate disadvantaged groups, reinforcing class disparities. When expansion is broader and accompanied by equity-oriented policies, inequality tends to diminish, although it rarely disappears.

Taken together, these hypotheses provide complementary analytical frameworks for assessing how system coverage and institutional organization shape inequalities of educational opportunity. Their empirical relevance depends on each country's historical and structural conditions. In Argentina and Chile, where expansion occurred in contexts of high social inequality and institutional segmentation, evidence does not show a sustained reduction in inequality, in contrast to what has been observed in some European countries or the United States (Boniolo et al., 2025a; Solís, 2018).

In Argentina, the public and tuition-free system expanded access, yet inequalities remain concentrated in secondary completion and university graduation, where household cultural capital and territorial disparities continue to shape outcomes. In Chile, expansion under a market-based regime deepened institutional segmentation and reinforced social selectivity. In Mexico, the coexistence of public and private sectors led to broader coverage, accompanied by strong regional and quality disparities.

Building on these hypotheses, the theoretical approach adopted here assumes that social class of origin continues to organize educational trajectories even under conditions of system expansion. Household occupational position reflects a set of economic, social, and symbolic resources that shape individuals' capacity to navigate educational transitions. In this sense, massification does not sharply reduce pre-existing inequalities, but rather reconfigures their expression across transitions. Household educational climate constitutes a specific dimension of social origin, linked to the intergenerational transmission of dispositions, practices, and educational expectations. Parental education affects academic support conditions and family strategies vis-à-vis schooling, even when material resources are controlled. Accordingly, household educational climate is expected to exert an effect that is not reducible to occupational class.

The temporal dimension is incorporated under the assumption that educational opportunities do not remain constant over time. Cohorts reflect historically situated positions shaped by system expansion, institutional reforms, and labor-market conditions. As a result, the relationship between social origin and educational attainment may vary across cohorts. Gender is also included as a constitutive dimension of educational inequality and an indicator of system expansion over time. Educational trajectories differ for men and women, and observed gaps depend both on educational level and the historically situated institutional context.

In addition, several studies have underscored the role of ethnic origin in processes of educational stratification, thus contributing to intersectional perspectives. In Argentina, the findings of Boniolo et al. (2025b) indicate that ethnic origin plays a significant role in the completion of compulsory education, although its effect diminishes as individuals progress to post-compulsory levels. By contrast, in the Mexican case, ethnic origin appears to exert a stronger influence on the transition from secondary to higher education (Rodríguez, 2018), while also contributing to the segmentation of educational opportunities for subaltern ethnic groups (Solís and Blanco, 2018). A similar pattern, albeit with lower intensity, has been documented in Chile (Núñez, 2017)^1^.

In sum, educational expansion is approached as an ambivalent process. Far from necessarily implying a reduction in inequality, it may coexist with the persistence—or even the reconfiguration—of origin-based disparities. Increased coverage does not guarantee equalization of opportunity; rather, it may relocate inequality across stages, dimensions, or pathways within the educational trajectory. From a structural perspective, the absorptive capacity of higher education plays a central role in determining where inequalities concentrate. In systems with restricted coverage, disparities tend to cluster at earlier stages, particularly in secondary completion. When expansion is broader, inequality tends to shift toward later phases of the educational trajectory.

## 4 Data and methods

The study adopts a comparative research design to analyze educational transitions in three Latin American countries—Argentina, Chile, and Mexico—using nationally representative probability surveys. The methodological objective is to assess how social stratification factors affect the likelihood of completing each educational level (primary, secondary, and tertiary) and transitioning to the next stage, taking into account both structural constraints and institutional differences across educational systems.

For cross-national comparability, educational levels were harmonized according to the UNESCO International Standard Classification of Education (ISCED 2011). This harmonization is feasible due to the regional similarity in Latin American educational systems, which share a sequence of compulsory levels (primary and secondary) and have experienced sustained expansion in higher education. Table 1 shows this situation.

**Table 1:** Standardization of education system levels by country.

Level analyzed in the study	Argentina	Chile	México	Equivalence in the international classification (ISCED-2011)
Primary education	Primary education (Grades 1° to 6° or 7°)	Basic education—First cycle (1st to 6th grade).	Primary education (Grades 1° a 6°).	Level 1—Primary Education
Lower secondary education	Basic cycle of secondary level (1st to 3rd year).	Basic education—second cycle (7th and 8th grade).	Secondary Level (1st to 3rd year).	Level 2—Lower secondary education.
Higher secondary education	Specialized cycle (Years 4–6).	Secondary education (Grades 9–12).	High school (grades 4–6 of upper secondary education).	Level 3—(Upper secondary education)
Higher education (university or tertiary)	National universities, private universities and non-university tertiary institutes.	Traditional and private universities, and professional and technical institutes.	Public and private universities and technological institutes.	Levels 5–6—Tertiary education (short or university).

For Argentina, we use the National Survey on Social Structure and Public Policies during the COVID-19 Pandemic (ESAyPP/PISAC-COVID-19, 2021), a nationally representative probability survey of the urban population. For Chile, we rely on the National Public Opinion Survey conducted by the Center for Public Studies (CEP, 2019), which includes a module on education and intergenerational mobility. For Mexico, we use the Intergenerational Social Mobility Module (INEGI, 2016), specifically designed to examine the relationship between social origin and educational attainment.

This study presents several limitations that should be considered when interpreting its results, stemming from the use of secondary data and its comparative analytical scope. First, although the surveys used are not strictly contemporaneous—Mexico (2016), Chile (2019), and Argentina (2021)—they fall within a relatively close time frame around 2020. These temporal differences reflect constraints associated with the use of available secondary data rather than substantive analytical choices. The study focuses on medium- and long-term structural processes of educational stratification, which are less sensitive to short-term fluctuations and thus remain comparable across these points in time. Nevertheless, contextual changes may have occurred between survey years. Argentina's data were collected during the COVID-19 pandemic, potentially affecting educational and socioeconomic conditions. However, the analysis is restricted to individuals aged 25–40, whose educational trajectories were largely completed prior to the pandemic, suggesting that any resulting bias is likely limited (Alcoba, 2014; Dalle et al., 2019; García de Fanelli, 2014; Jorrat, 2010). Second, the use of a reduced three-class version of the EGP schema entails a loss of detail in capturing within-class differences, particularly in contexts of high occupational heterogeneity; this operational decision is mainly driven by sample size constraints and the need to enable comparisons beyond Latin America. Third, the analysis relies on surveys that do not allow the inclusion of relevant unobserved variables, such as prior academic performance or institutional characteristics, limiting the ability to capture finer stratification mechanisms. Finally, the binary operationalization of gender reflects data limitations and does not account for non-binary identities or more complex intersectional experiences, although their representation in surveys is typically low. These limitations open avenues for future research.

Descriptive figures were produced for populations aged 25–65 to provide an overall picture of each country, while the multivariate models focus on individuals aged 25–40 in order to capture *completed educational trajectories* and ensure intergenerational comparability. This age restriction also guarantees an adequate number of observations for robust statistical inference. The analysis is sequential: for each transition, the population at risk consists only of individuals who completed the previous level.

We analyze four educational transitions: (1) entry into upper secondary education, (2) completion of upper secondary education, (3) access to tertiary education, and (4) completion of tertiary education. For each country, we estimate both descriptive indicators (completion and access rates in the general population) and conditional models that assess the probability of advancing to the next level given that the previous one was completed.

The independent variables include social class of origin, sex, and household educational climate. The variables included in the models correspond to different analytical levels. Social class of origin and household educational climate constitute the core explanatory variables, as they capture the structural position and cultural dimension of social origin that shape educational opportunities across trajectories. Social class reflects family labor market insertion, while household educational climate refers to the intergenerational transmission of dispositions, expectations, and educational support. Gender is incorporated as a constitutive dimension of educational inequality and as an indicator of the differentiated effects of educational expansion on women and men in Latin America. Finally, birth cohorts are included as temporal control variables to capture changes associated with educational expansion over time.

Social class of origin was constructed using an adapted three-class version of the EGP scheme (Erikson et al., 1979), based on its Latin American operationalization (Solís and Boado, 2016). Due to sample-size constraints, we use three macro-categories: service class, intermediate class, and working-class. The binary operationalization of gender responds to empirical constraints imposed by the data sources, as some of the surveys used distinguish only between men and women. This limitation prevents the empirical capture of non-binary identities, performative dimensions of gender, or more complex intersectional experiences within a quantitative comparative research design. However, the analysis does not treat gender as a homogeneous or autonomous axis of inequality; that is, gender does not have an inherent effect but rather a socially constructed one. On the contrary, the results reveal heterogeneous and context-specific patterns. Household educational climate was operationalized as the average educational level of the parents and grouped into three categories: high (some tertiary education or more), medium (completed secondary education), and low (incomplete secondary or less).

Separate logistic regression models were estimated for each country and educational transition. From these models, average marginal effects (AMEs) were computed to estimate the average impact of each independent variable on the probability of transition. AMEs are particularly useful for comparison across groups and models with non-linear functional forms (Ballesteros, 2018).

To assess potential collinearity, we examined the relationship between social class of origin—measured through the occupation of the main household earner—and household educational climate, defined as the average level of parental education. Although both variables capture different dimensions of social origin, they were moderately correlated, with no evidence of multicollinearity that would compromise model stability. Social class reflects individuals' structural position in the labor market, whereas household educational climate captures the cultural dimension of family background. The simultaneous inclusion of both variables thus allows us to disentangle the economic and cultural mechanisms underlying inequality in educational transitions. While the surveys are not perfectly contemporaneous, the focus on long-term structural differences supports their analytical comparability.

## 5 Results

### 5.1 The expansion of educational systems in Argentina, Chile, and Mexico

**Figure 1:**
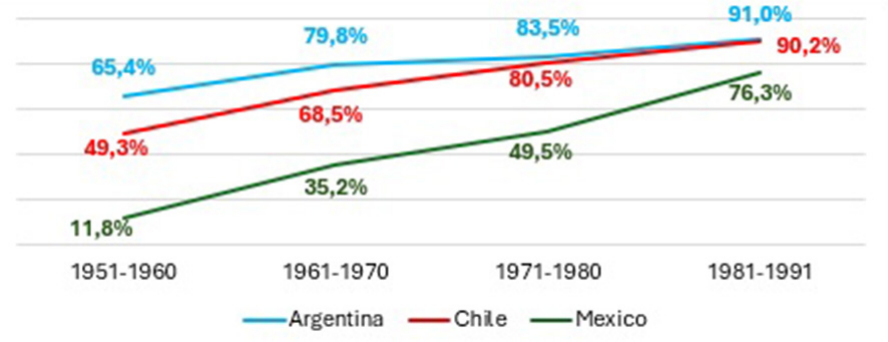
Access to secondary education according to birth cohorts in Argentina, Chile and Mexico. Prepared by the author based on ESAyPP/PISAC-COVID-19 (2021), ENOP- CEP (2019), MMSI- INEGI (2016).

**Figure 2:**
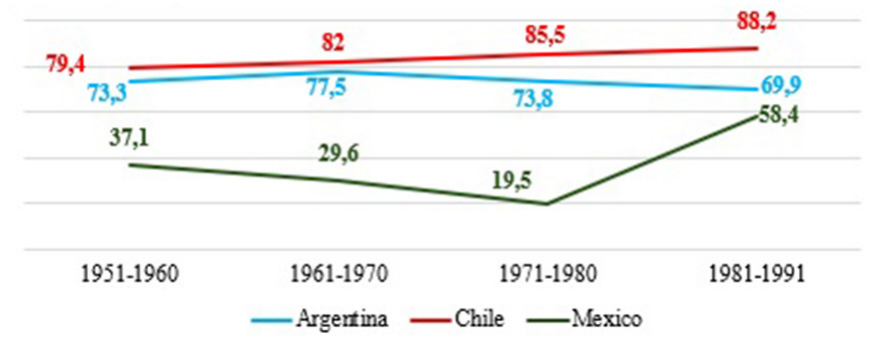
Secondary school graduation rates according to birth cohorts in Argentina, Chile, and Mexico. Prepared by the author based on ESAyPP/PISAC-COVID-19 (2021), ENOP- CEP (2019), MMSI- INEGI (2016).

**Figure 3:**
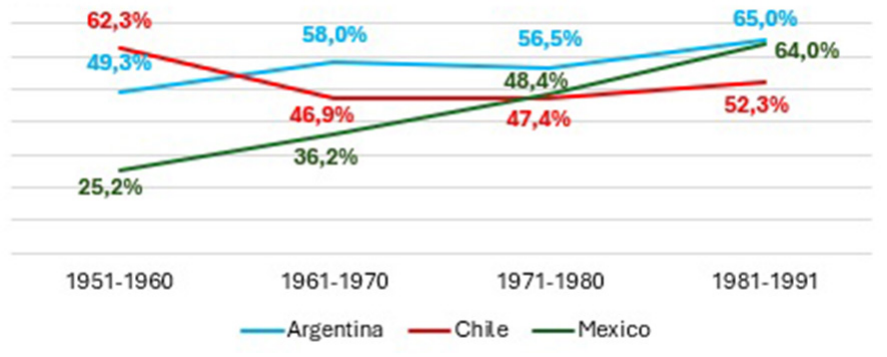
Access to higher education according to birth cohorts in Argentina, Chile and Mexico. Prepared by the author based on ESAyPP/PISAC-COVID-19 (2021), ENOP- CEP (2019), MMSI- INEGI (2016).

**Figure 4:**
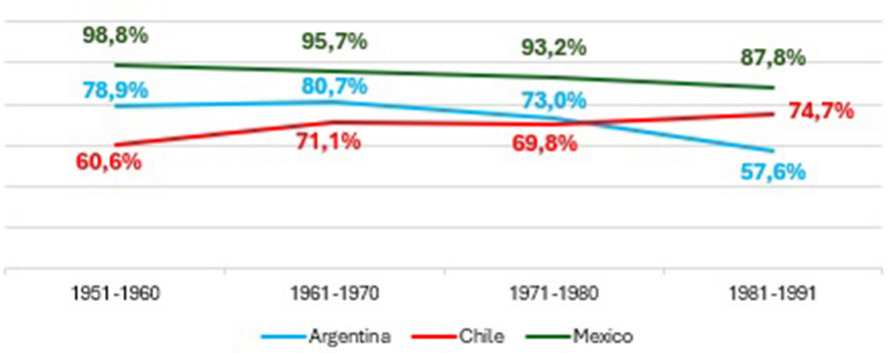
Higher education graduation rates according to birth cohorts in Argentina, Chile, and Mexico. Prepared by the author based on ESAyPP/PISAC-COVID-19 (2021), ENOP- CEP (2019), MMSI- INEGI (2016).

This section examines the evolution of educational expansion in Argentina, Chile, and Mexico, focusing on the progression from access to upper secondary education to graduation from higher education. Figures 1–4 present trends across birth cohorts from 1921 to 1991, allowing us to identify long-term patterns of incorporation, retention, and completion within each educational system.

Figure 1 shows de acces to secundary school. All three countries show a sustained increase in access to upper secondary education across successive cohorts. Argentina and Chile approach full incorporation into the secondary level, while Mexico follows the same trajectory with a slight delay but still reaches comparatively high levels. These patterns indicate that access to secondary schooling has become generalized across the region, although through distinct institutional pathways.

Figure 2 reveals marked cross-national differences in the consolidation of secondary completion. Chile exhibits the most consistent pattern: the cohort born between 1951 and 1960 already shows high graduation rates, which continue to rise steadily and surpass 88% in the 1981–1991 cohort. This suggests an early and effective universalization of upper secondary education, with strong system capacity to retain growing student populations. Argentina follows a less linear trajectory. Although graduation levels remain comparatively high, the increase across cohorts is modest and followed by a decline in the youngest cohort. Individuals born between 1981 and 1991 have lower completion rates than those in previous cohorts, suggesting that massification was not accompanied by institutional mechanisms capable of supporting full completion. Mexico shows a discontinuous pattern. The earliest cohort exhibits low levels of secondary completion, reflecting a later expansion of the secondary level. This is followed by an additional decline and then a sharp recovery in the 1981–1991 cohort. Even so, Mexico remains below Argentina and Chile in all cohorts, highlighting a delayed and still incomplete universalization of secondary education.

Taken together, these trajectories show that secondary expansion adopted distinct institutional configurations. Chile consolidated the level early; Mexico advanced later and unevenly; and Argentina expanded access but faced persistent challenges in retention and completion. Secondary graduation remains a critical point of stratification across the three systems.

Access to higher education also shows diverging paths in Figure 3. Argentina experiences a sustained increase—from 49.3% in the 1951–1960 cohort to 65% in the 1981–1991 cohort—interrupted only by a decline during the last military dictatorship, after which growth resumed. Mexico presents the most accelerated and continuous rise, moving from very low levels toward values close to those of Argentina, reflecting a late but steady expansion of higher education. The Chilean case deserves special attention due to its non-linear pattern. While relatively high levels of access to higher education are observed among older cohorts, there is a decline in intermediate cohorts, followed by a partial recovery among younger ones. This contrasts with the more sustained expansion in Argentina and the steadier upward trend in Mexico. Chile's trajectory reflects a highly segmented, market-oriented higher education system, where access has been shaped by financing mechanisms, institutional differentiation, and entry costs. Consequently, variations in access are driven not only by expansion in coverage but also by shifts in access rules and system structure, explaining its discontinuous evolution.

Graduation trends highlight additional contrasts in Figure 4. Mexico shows the highest tertiary graduation rates across cohorts, despite a decline in the most recent one. These high levels suggest that access to higher education remains strongly selective and that those who enter have a high probability of completing their studies. This points to a system with strong filtering mechanisms prior to entry. Chile exhibits a gradual upward trend, indicating consolidation of a diversified tertiary sector—including professional and technical programs—that has expanded the system's capacity to retain and graduate students from varied social backgrounds. Argentina displays a declining trend in tertiary graduation, particularly in the youngest cohort. This pattern suggests that expansion has been accompanied by growing difficulties in completion. The rapid enlargement of the university system increased access, but the system has yet to develop sufficient institutional capacity to support higher completion rates.

Across the three countries, the expansion of access did not translate into a proportional expansion of successful completion. The distinction between *access* and *effective attainment* becomes clear: while more individuals enter secondary and higher education, significant barriers remain at the point of graduation, especially in Mexico for secondary completion and in Argentina for completion of higher education. These findings highlight the central role of system design, institutional capacity, and retention mechanisms in shaping educational outcomes in Latin America.

### 5.2 A comparative analysis of educational transitions

Table 2 presents a first descriptive overview of educational transitions in the three countries under study. The table identifies critical turning points along educational trajectories by indicating the stages at which losses in educational opportunities become apparent. To this end, it reports completion and entry rates from primary schooling through access to higher education. In addition, transition rates are calculated conditional on having completed the preceding educational level.

**Table 2:** Educational transitions of people aged 25–40.

Country	Start secondary (%)	Finish secondary (%)	Start superior (%)	Finish superior (%)
Argentina	90.5	75.1	61.5	50.1
Chile	95.9	88.8	51.5	70.9
México	80.6	36.9	54.3	78.7

Model conditioned on completion of the previous level. In percentages. Prepared by the author based on ESAyPP/PISAC-COVID-19 (2021), ENOP- CEP (2019), MMSI- INEGI (2016).

First, completion of primary education is virtually universal in all three countries, with rates above 96%. However, historical evidence shows that this process followed distinct national paths. Argentina achieved early universalization in the early twentieth century through a strongly state-led educational model aimed at national integration. In Chile, universalization unfolded in two stages: the first covering the initial four years of schooling during the 1920s and the second extending compulsory education to six years by the 1930s. Mexico represents a slower case, with near-universal primary schooling being reached only toward the 1990s (de Olguín Meza, 2018).

Differences across countries become visible at the transition into secondary education. While access has approached universality in Argentina and Chile, it remains a major constraint in Mexico. One plausible explanation is the delayed universalization of primary schooling, which limited the size of cohorts eligible to enter secondary education. Additionally, the introduction of compulsory secondary education in Mexico occurred relatively late, in 2012, compared to Argentina, where compulsory secondary education was established in 2006 under the National Education Law (No. 26,206), and Chile, which implemented it in 2003.

Completion of secondary education constitutes a major bottleneck in educational trajectories, particularly in Mexico and, to a lesser extent, in Argentina, whereas Chile displays comparatively lower attrition. In Mexico, only 36.9% of those who enter secondary school complete it and are therefore eligible to access higher education. This is consistent with previous research showing that rapid expansion occurred without sufficient institutional capacity to retain students, resulting in high dropout, repetition, and educational delay (Blanco Bosco, 2011). These problems are especially pronounced in upper secondary education (“Educación Media Superior”), where enrollment reaches only 65% among adolescents aged 15–18, combined with low achievement in language and mathematics by regional standards (de Olguín Meza, 2018; Solís, 2013).

Argentina also shows regressive effects of secondary expansion: increased enrollment has not been matched by sustained improvements in graduation rates. This issue is further aggravated by widespread educational delay (Boniolo and Najmias, 2018) and uneven academic quality across institutions (Boniolo and Lemos, 2025). Chile presents the lowest dropout rates between enrollment and graduation, although its expansion has stagnated in recent decades despite its larger education system relative to Argentina.

Access to higher education marks the sharpest inequality for Chile, especially when contrasted with Argentina. While 61.5% of Argentine secondary graduates enter higher education, the corresponding figures fall to 54.3% in Mexico and 51.5% in Chile. In Chile, these patterns likely reflect the late introduction of tuition-free higher education and sustained institutional segmentation driven by market-oriented reforms (Cabrera, 2016; Solís and Dalle, 2018). In contrast, Argentina's system has been tuition-free since 1949 and expanded mainly through publicly funded universities located in urban working-class areas, explicitly aimed at incorporating first-generation students (Boniolo et al., 2025b).

Interestingly, the pattern reverses at the point of higher education completion. Argentina exhibits the lowest graduation rates among those who entered tertiary education, followed by Chile and then Mexico. Specifically, only about half of Argentines aged 25–40 who entered higher education obtained a degree. This highlights limited absorptive capacity under rapid expansion, resulting from the combined effects of compulsory secondary education and massive growth in tertiary enrollment. Previous studies emphasize that secondary education often fails to provide adequate preparation for tertiary curricula, while early labor market entry constrains educational continuity, particularly in the first years of higher education (Jacinto, 2013; Miranda, 2006; Sosa and Lemos, 2025). By contrast, Chile and Mexico display stronger social selection at entry into higher education (Mare, 1980) and, consequently, higher completion rates among those who do enroll.

In sum, the descriptive analysis indicates that educational expansion has not translated into greater equity in educational transitions. Inequality in Mexico emerges early, both in access to and completion of secondary education. In Chile and Argentina, disparities are concentrated at the tertiary level: in the former, in access; in the latter, in persistence and graduation. These findings confirm that expansion has generated differentiated national trajectories shaped by institutional arrangements and policy designs.

### 5.3 Social factors in educational transitions

This section presents marginal effects models to estimate the likelihood of successfully completing educational transitions. Each model is estimated among individuals who meet the precondition for the transition under analysis: having completed the previous level or having entered the level whose completion is being modeled. Table 3 shows this model. Although social origin and educational climate remain statistically significant in the estimated models, the interaction analysis indicates that their effects are not parallel but conditional (Table 4). These interactions do not suggest collinearity, but rather substantive interdependence. Given the objective of estimating comparable effects consistent with the regional literature, the additive model is prioritized, as it allows for the identification of the net contribution of each dimension. While interactions are important for capturing specific configurations of inequality, they are presented in the appendix as complementary analyses. This strategy consolidates the results without compromising the interpretive clarity of the main model.

**Table 3:** Average marginal effects on the chances of passing the steps of the educational transitions of people aged 25–40.

		Educational transition			
Country	Independent variables	Start secondary	Finish secondary	Start superior	Finish superior
ARGENTINA	Social class of origin [Ref. Services]				
	Intermediate	−0.076^***^	−0.072^*^	−0.143^*^	−0.030+
	Working class	−0.155^***^	−0.206^***^	−0.088^***^	−0.190^***^
	Sex [Ref.Women]				
	Man	−0.002+	−0.049^*^	−0.101^***^	−0.088^**^
	Educational climate of the home [Ref. High]				
	Medium	−0.028^*^	−0.024+	−0.217^***^	−0.146+
	Low	−0.180^***^	−0.205^***^	−0.308^***^	−0.312^***^
	*R*2	0.148	0.128	−0.107	0.157
	Number of cases	(3.899)	(3.348)	(2.480)	(768)
CHILE	Social class of origin [Ref. Services]				
	Intermediate	−0.147+	−0.011+	−0.236^**^	−0.011
	Working class	−0.168+	−0.043+	−0.350^***^	−0.104^*^
	Sex [Ref. Women]				
	Men	−0.053+	−0.019+	−0.037+	−0.018+
	Educational climate of the home [Ref. High]				
	Medium	−0.130+	−0.094^***^	−0.226^***^	−0.069+
	Low	−0.270^***^	−0.187^***^	−0.402^***^	−0.179^**^
	*R*2	0.215	0.108	0.181	0.140
	*N*	(925)	(709)	(610)	(359)
MÉXICO	Social class of origin [Ref. Services]				
	Intermediate	−0.045^***^	−0.111^***^	−0.365^***^	−0.098^**^
	Working class	−0.291^***^	−0.310^***^	0.710^***^	−0.148^***^
	Sex [Ref. Women]				
	Men	0.015^*^	−0.084^***^	0.002+	−0.056^***^
	Educational climate of the home [Ref. High]				
	Medium	−0.027^**^	−0.085^**^	−0.102+	−0.046^**^
	Low	−0.374^***^	−0.388^***^	−1,112^***^	−0.186^***^
	*R*2	0.107	0.118	0.101	0.090
	*N*	(19.347)	(14.328)	(7.710)	(2.254)

Model conditioned on having completed the previous step. Prepared by the author based on ESAyPP/PISAC-COVID-19 (2021), ENOP- CEP (2019), MMSI- INEGI (2016).

^***^p < 0.001;

^**^p < 0.01;

^*^p < 0.05,

+ Not consider.

**Table 4:** Average marginal effects on the chances of passing the steps of the educational transitions of people aged 25–40.

Country	Independent variables	Educational transition			
		Start secondary	Finish secondary	Start superior	Finish superior
ARGENTINA	Social class of origin x educational climate of the home [Ref. Services with high level]				
	Services with medium level	−0.004+	−0.146+	−0.308+	−0.516+
	Services with low level	−0.059+	−0.163+	−0.376+	−0.586+
	Intermediate with high level	−0.019+	−0.327^*^	−0.230+	−0.483^*^
	Intermediate with medium level	−0.052^*^	−0.181^**^	−0.201+	−0.566^**^
	Intermediate with low level	−0.159^*^	−0.281^**^	−0.541+	−0.572^**^
	Working Class with high level	–	-	-	-
	Working Class with medium level	−0.141^**^	−0.319^*^	−0.544^*^	−0.324^***^
	Working Class with low level	−0.249^***^	−0.446^***^	−0.628^***^	−0.514^***^
	Sex [Ref. Women]				
	Men	−0.002+	−0.049^*^	−0.107^**^	−0.093^**^
	*R*2	0.045	0.079	0.098	0.086
	Number of cases	(3.899)	(3.348)	(2.480)	(768)
CHILE	Social class of origin x educational climate of the home [Ref. Services with high level]				
	Services with medium level	−0.124+	−0.111+	−0.319^*^	−0.150+
	Services with low level	-	-	-	-
	Intermediate with high level	−0.099+	−0.092^*^	−0.228^***^	−0.577^*^
	Intermediate with medium level	−0.251+	−0.177^*^	−0.470^***^	−0.487^*^
	Intermediate with low level	−0.301+	−0.250^**^	−0.383^***^	−0.107+
	Working Class with high level	-	-	-	-
	Working Class with medium level	−0.033+	−0.110^**^	−0.616^***^	−0.545^*^
	Working Class with low level	−0.135+	−0.187^**^	−0.716^***^	−0.719^***^
	Sex [Ref. Women]				
	Men	−0.062+	−0.022+	−0.050+	−0.021+
	*R*2	0.097	0.066	0.182	0.103
	*N*	(925)	(709)	(610)	(359)
MÉXICO	Social class of origin x educational climate of the home [Ref. Services with high level]				
	Services with medium level	−0.003+	−0.026+	−0.174+	−0.180+
	Services with low level	−0.004+	−0.214^*^	−0.365^**^	−0.253^*^
	Intermediate with high level	−0.008+	−0.107^***^	−0.259^***^	−0.225^**^
	Intermediate with medium level	−0.028+	−0.196^***^	−0.352^***^	−0.227^***^
	Intermediate with low level	−0.115^***^	−0.368^***^	−0.526^***^	−0.303^***^
	Working Class with high level	−0.110+	−0.148^***^	−0.344^***^	−0.302^**^
	Working Class with medium level	−0.145^***^	−0.240^***^	−0.422^***^	−0.213^**^
	Working Class with low level	−0.240^***^	−0.569^***^	−0.628^***^	−0.269^**^
	Sex [Ref. Women]				
	Men	0.012^*^	0.098^***^	0.014+	−0.052^**^
	*R*2	0.090	0.126	0.098	0.071
	*N*	(19.347)	(14.328)	(7.710)	(2.254)

Model conditioned on having completed the previous. Interaction between social class of origin and educational climate of the home.

*** *p* < 0.001;

** *p* < 0.01;

* *p* < 0.05, + Not consider.

A comparative perspective is adopted to identify both regional regularities and national specificities in the effects of social origin, family educational background, and gender on educational transitions in Argentina, Chile, and Mexico.

The first analytical stage refers to access to secondary education and shows negative effects for the intermediate and working classes in all three countries, although with different intensities. While Argentina and Mexico exhibit statistically significant disadvantages for both the intermediate and especially the working class, social origin does not emerge as a relevant predictor in Chile. Specifically, individuals from working-class backgrounds in Argentina have 15.5 percentage points lower probabilities of entering secondary education, and this penalty nearly doubles in Mexico (29.1%).

Gender effects also differ across countries. In Argentina, women display higher probabilities of entering secondary education, whereas in Mexico the advantage favors men. Family educational background is the only variable that is statistically significant in all three countries. Low-education households are associated with the largest penalties in Mexico (−37.4 percentage points), followed by Chile (−27%) and Argentina (−18%). Notably, in Chile family educational background is the only significant variable in this transition, and its effect clearly exceeds that of social class.

Completion of secondary education exhibits patterns similar to those observed at entry, although some effects become more pronounced. In Chile, only family educational background remains statistically significant, although its impact is weaker compared to the previous transition. In Argentina, class inequality increases, while in Mexico gender gaps become more pronounced. Individuals from working-class backgrounds experience stronger disadvantages in Mexico (−31 percentage points) than in Argentina (−20.6). Although Mexico shows the largest penalty, Argentina stands out because the class gap increases more sharply between secondary entry and completion than in any other country.

Gender differences also intensify. In Argentina, men exhibit a disadvantage of 4.9 percentage points in secondary completion, whereas in Mexico men display a positive differential of 8.4 percentage points. These effects are weakly significant in Argentina but highly significant in Mexico. Regarding family background, Chile shows a decline in the negative effect of low parental education: the penalty decreases from around 27% at entry into secondary education to 18.7% at completion. In contrast, penalties remain high in Argentina (−20.5%) and Mexico (−38.8%). Overall, and consistent with the descriptive findings, Mexico is the country where secondary education constitutes the most restrictive bottleneck.

Turning to higher education, gender effects persist in both access and completion. While no significant differences are observed in Chile, men face stronger barriers in Argentina—especially at the entry stage—whereas in Mexico women experience lower probabilities of completing tertiary education once enrolled.

Access to higher education constitutes the sharpest point of stratification in Chile. Individuals from working-class backgrounds have 35 percentage points lower probabilities of entering tertiary education, and this penalty increases to 40.2% among those from low-education households. Mexico shows even stronger disparities: the intermediate class exhibits a disadvantage of 36.5 percentage points, while working-class individuals face a penalty of 71%. Low parental education is also a crucial dimension in Mexico, where individuals from low-education households have twice the probability of not entering higher education compared to those from highly educated families.

Argentina appears as a partial exception. Although educational background remains influential, the effects of social class on access are considerably weaker. Individuals from working-class households show only an 8.8 percentage point disadvantage in entering tertiary education, which represents a substantial decline relative to the penalty observed at the end of secondary school.

The final stage—completion of higher education—reveals additional patterns. In Chile and Mexico, class inequalities decrease markedly, whereas in Argentina penalties remain comparatively higher. Disadvantages for working-class individuals reach 10% in Chile (with weak statistical significance), 14.8% in Mexico, and 19% in Argentina, where the effect remains strongly significant. A similar pattern emerges for family educational background, with stable penalties in Argentina and notable reductions in the other two countries.

These results suggest that countries face difficulties in converting secondary completion into tertiary degrees, but the main bottleneck differs across contexts. In Chile and Mexico, inequalities are concentrated at the point of entry into higher education, consistent with the hypothesis of social selection (Mare, 1980). In Argentina, by contrast, inequalities are more salient in persistence and graduation, which aligns with the logic of maximal maintained inequality articulated by Hout and Rafftery (1993).

## 6 Discussion

The findings indicate that educational expansion in Argentina, Chile, and Mexico did not translate into a substantive equalization of educational opportunities. Although access to educational levels expanded, ascriptive factors continue to structure trajectories decisively, generating persistent inequality patterns whose form varies depending on national institutional contexts.

From a theoretical standpoint, the evidence does not support a linear interpretation of expansion as an inherently democratizing process. Inequalities do not systematically decline at higher levels, nor is a homogeneous convergence observed across countries. In this sense, the results depart from the expectations derived from the hypothesis of declining selectivity (Mare, 1980) and from a mechanical interpretation of saturation (Hout and Rafftery, 1993). Instead, the evidence aligns more clearly with the frameworks of effectively maintained inequality (Lucas, 2001) and inequality linked to coverage (Solís, 2013, 2018; Solís, 2019), both of which emphasize the reorganization rather than the elimination of inequality in contexts of expansion.

The comparative analysis confirms that educational systems are not neutral structures, but mechanisms that allocate opportunities differentially according to their institutional designs. In Chile, segmentation and selection mechanisms intensify entry barriers at the tertiary level. In Mexico, structural limitations at the secondary level operate as an early filter that shapes subsequent trajectories. In Argentina, although tertiary access is comparatively broad, constraints emerge mainly in persistence and graduation. These patterns demonstrate that the form inequality takes is deeply dependent on the educational regime in which trajectories unfold.

Furthermore, the results highlight the need to distinguish between structural and cultural dimensions of social origin. While occupational class continues to shape differential access, family educational background operates as an autonomous mechanism of intergenerational transmission, influencing educational expectations, household strategies, and support conditions. This dual structure contributes to the cumulative reproduction of advantage and disadvantage.

Regarding the gender variable, comparative research shows that women tend to achieve better academic performance and exhibit higher levels of engagement from early stages, which translates into greater probabilities of educational progression in both secondary and higher education (Buchmann et al., 2008). This pattern is strongly confirmed in the case of Argentina and is linked not only to cognitive performance but also to non-cognitive skills, school behaviors, and educational expectations that support the continuity of female trajectories (DiPrete and Buchmann, 2013). In Mexico, the findings point to more complex configurations, including male advantages in completing secondary education and female disadvantages in graduating from higher education. These disparities are associated with persistent structural inequalities in educational and labor market opportunities, as well as family care dynamics, prolonged educational trajectories, and gendered expectations (Blanco Bosco, 2011; Rodríguez, 2020). In Chile, the absence of significant gender effects in several transitions can be understood within a highly segmented educational system, where social origin and institutional characteristics more strongly shape opportunities. In such contexts, socioeconomic inequalities tend to outweigh other dimensions, including gender (Mizala and Torche, 2017).

From a broader Latin American perspective, educational expansion has reduced gender gaps at lower levels and, in some cases, generated female advantages, but it has not eliminated the persistent influence of social origin or inequalities at higher levels (Torche, 2010). Thus, gender should be understood as a dynamic dimension that is reconfigured across educational transitions.

Overall, educational expansion in the region constituted a socially stratified process. Absolute access increased, but the likelihood of completing educational trajectories and obtaining socially valued credentials remained strongly patterned by family background. These findings suggest that policies focused exclusively on expanding coverage are insufficient and that more targeted interventions are required, particularly in access mechanisms, student retention, and the institutional mediation between social origin and educational achievement.

## 7 Conclusions

By the end of the second decade of the twenty-first century, educational expansion in Argentina, Chile, and Mexico had increased access but had not substantially reduced relative inequalities between social classes. Educational trajectories remain structured by ascriptive characteristics—especially social class and family educational background—although the expression of inequality varies according to institutional design.

The findings indicate that educational expansion does not operate in a homogeneous manner; rather, it is articulated with national institutional configurations, generating differentiated patterns of stratification. In Chile, post-2011 educational reforms were implemented within a highly segmented system, thereby reinforcing forms of horizontal stratification across institutions, in line with the Effectively Maintained Inequality hypothesis. In Argentina, policies aimed at expanding secondary school completion (such as AUH, Plan FINES, and Progresar) and at facilitating access to higher education through the creation of new national universities with introductory leveling courses contributed to lowering entry barriers; however, they failed to dismantle inequalities in persistence and graduation. This suggests limits to institutional absorptive capacity and a displacement of inequality mechanisms toward later stages of the educational trajectory. In contrast, in Mexico, the main bottlenecks are concentrated in earlier phases of the educational pathway, associated with the late incorporation of compulsory schooling and persistent territorial inequalities, thereby reinforcing processes of social selectivity prior to access to higher education.

On the other hand, even if it has an effect in our models, gender does not operate in a uniform manner; rather, according to the literature, it interacts with labor markets, educational institutions, and family strategies within each national context. For instance, the male disadvantages observed in Argentina are interpreted in relation to early labor market entry and weaker institutional mechanisms for educational retention (Jacinto, 2013). In Mexico, gender differences are discussed in light of stronger selection processes and differentiated labor market expectations (Blanco Bosco, 2011). In Chile, the absence of significant gender effects is interpreted within a context in which institutional segmentation and the weight of family educational background tend to overshadow gender differences (Mizala and Torche, 2017).

Family educational background continues to operate as a central mechanism of intergenerational transmission, independent of occupational class. This cultural dimension reinforces cumulative disadvantage even under expanded access. Gender, in turn, does not represent a uniform axis of inequality but varies across contexts and educational stages.

From a theoretical perspective, the evidence supports a non-linear conception of expansion as a democratizing process and aligns with approaches that emphasize the reconfiguration of inequality under massification, particularly effectively maintained inequality and inequality linked to coverage.

In sum, equality of opportunity depends not only on formal access, but also on the ability to complete educational trajectories and obtain credentials with labor-market value and personal significance. The comparison between Argentina, Chile, and Mexico highlights the need for policies that combine expansion with sustained equity strategies, particularly focused on the most critical transition points in each system.

## Data Availability

The datasets presented in this study can be found in online repositories. The names of the repository/repositories and accession number(s) can be found below: https://www.argentina.gob.ar/ciencia/pisac/bases-de-datos.

## Footnotes

1 In this study, the ethnic dimension is not included in the research design due to the absence of a comparable variable for the Chilean case. We also emphasize the need to systematically integrate ethnoracial variables into sociological surveys in Latin America.
